# Up‐regulation of miR‐195 contributes to cardiac hypertrophy‐induced arrhythmia by targeting calcium and potassium channels

**DOI:** 10.1111/jcmm.15431

**Published:** 2020-05-28

**Authors:** Lina Xuan, Yanmeng Zhu, Yunqi Liu, Hua Yang, Shengjie Wang, Qingqi Li, Chao Yang, Lei Jiao, Ying Zhang, Baofeng Yang, Lihua Sun

**Affiliations:** ^1^ Department of Pharmacology Harbin Medical University (the State‐Province Key Laboratories of Biomedicine‐Pharmaceutics of China, Key Laboratory of Cardiovascular Research, Ministry of Education) College of Pharmacy Harbin Medical University Harbin, Heilongjiang China

**Keywords:** arrhythmia, cardiac hypertrophy, Cavβ1, Kir2.1, Kv4.3, miR‐195

## Abstract

Previous studies have confirmed that miR‐195 expression is increased in cardiac hypertrophy, and the bioinformatics website predicted by Targetscan software shows that miR‐195 can directly target CACNB1, KCNJ2 and KCND3 to regulate Cavβ1, Kir2.1 and Kv4.3 proteins expression. The purpose of this study is to confirm the role of miR‐195 in arrhythmia caused by cardiac hypertrophy. The protein levels of Cavβ1, Kir2.1 and Kv4.3 in myocardium of HF mice were decreased. After miR‐195 was overexpressed in neonatal mice cardiomyocytes, the expression of ANP, BNP and β‐MHC was up‐regulated, and miR‐195 inhibitor reversed this phenomenon. Overexpression of miR‐195 reduced the estimated cardiac function of EF% and FS% in wild‐type (WT) mice. Transmission electron microscopy showed that the ultrastructure of cardiac tissues was damaged after miR‐195 overexpression by lentivirus in mice. miR‐195 overexpression increased the likelihood of arrhythmia induction and duration of arrhythmia in WT mice. Lenti‐miR‐195 inhibitor carried by lentivirus can reverse the decreased EF% and FS%, the increased incidence of arrhythmia and prolonged duration of arrhythmia induced by TAC in mice. After miR‐195 treatment, the protein expressions of Cavβ1, Kir2.1 and Kv4.3 were decreased in mice. The results were consistent at animal and cellular levels, respectively. Luciferase assay results showed that miR‐195 may directly target CACNB1, KCNJ2 and KCND3 to regulate the expression of Cavβ1, Kir2.1 and Kv4.3 proteins. MiR‐195 is involved in arrhythmia caused by cardiac hypertrophy by inhibiting Cavβ1, Kir2.1 and Kv4.3.

## 1 INTRODUCTION

Cardiac hypertrophy can easily trigger atrial and ventricular arrhythmias, increase the risk of morbidity and mortality, and lead to sudden cardiac death in patients.^1^, ^2^ Long‐term or excessive stress load may lead to a transition from physiological hypertrophy to pathological hypertrophy, and eventually lead to heart failure (HF).^3^ HF patients are susceptible to develop arrhythmias, which represents the world's major medical burden.^4^, ^5^ The extended action potential duration (APD) at the end of hypertrophy is most likely due to a decrease in I_to_ density.^6^ According to reports, the Cav1.2 protein level and Ca^2+^ channel activity are reduced in cardiomyocytes with cardiac hypertrophy and HF caused by pressure overload.^7^ Decreased I_to_ and I_K1_ densities have also been reported in models of cardiac hypertrophy.^8^ These reduced I_to_ and I_K1_ might be the cause of prolonged action potential induced by hypertrophy. In cardiac hypertrophy and HF models, K^+^ and Ca^2+^ currents are down‐regulated and APD is prolonged, which showed significant electrophysiological remodelling. The I_to_ and I_K1_ are central regulators of arrhythmia and may be promising targets for anti‐arrhythmic approaches. However, the exact mechanisms regulating the decreased potassium and calcium channels in hypertrophy need further study.

In addition, many studies have shown that miRNAs play a key role in the regulation of ion channels.^9^, ^10^ It turns out that many miRNAs have been proved to be significantly changed in the development of arrhythmia by affecting different kinds of ion channels.^11^, ^12^ Moreover, so many miRNAs can be used as biomarkers for stress overload‐induced hypertrophy and cardiac electrical remodelling in HF.^13^, ^14^, ^15^ According to reports, miR‐195 plays an important role in regulating cardiovascular diseases. Inhibition of miR‐195 expression can increase Bcl‐2 expression and improve cardiomyocyte apoptosis induced by ischaemia‐reperfusion.^16^ Furthermore, miR‐195 inhibits v‐myb avian myeloblastosis viral oncogene homolog (MYB) transcription factors protein levels, thereby aggravating apoptosis induced by ischaemia‐reperfusion injury.^17^ After inhibiting miR‐195, coronary blood flow and myocardial function of diabetic patients induced by streptozotocin (STZ) were improved. In addition, miR‐195 can promote oxidative damage and angiogenesis and inhibit apoptosis.^18^ When miR‐195 is overexpressed, mice develop cardiac hypertrophy and cardiac dysfunction.^19^, ^20^ The miR‐195 is significantly elevated in mice with cardiac hypertrophy or heart failure by inhibiting high mobility group protein 1 (HMG1).^21^ In addition, miR‐195 expression is increased in α‐myosin heavy chain (α‐MHC) transgenic mice, and overexpression of miR‐195 inhibits recombinant human calbindin 39 (Cab39), thereby inhibiting adenosine 5’‐monophosphate (AMP)‐activated protein kinase (AMPK) signalling pathway, leading to hypertrophy.^22^ Therefore, current research indicates that miR‐195 can participate in the development of cardiac hypertrophy by acting on different targets. It plays an important role in cardiac hypertrophy, but the molecular mechanism of miR‐195 in arrhythmia caused by cardiac hypertrophy has not been explored, and the mechanistic basis of miR‐195 in regulating ion channel disorders in cardiac hypertrophy remains incompletely understood.

This research experiment objective is to explore the mechanistic basis underlying miR‐195 dysregulation in electrical remodelling and propose a possible novel interaction between miR‐195 and calcium, potassium channels.

## 2 MATERIAL AND METHODS

### 2.1 Construction of miR‐195 overexpression and AMO‐miR‐195 lentivirus vector

In this study, miR‐195 was constructed using the BLOCK‐iT polII miR‐RNAi expression vector and EmGFP kit, and construction of the vector was applied after plasmid sequence was analysed (Invitrogen). The final concentration of the constructed lentivirus is 1.0 × 10^9^ transducing U/mL for miR‐195 overexpression lentivirus vector. A lentivirus vector carrying miR‐195 inhibitor was constructed using the GV280 expression vector by Shanghai GeneChem Co., Ltd. The final concentration of the constructed lentivirus is 1.0 × 10^9^ transducing U/mL for miR‐195 inhibitor lentivirus vector. Virus suspensions were stored at −80°C, mixed and centrifuged on ice before use.

### 2.2 In vivo injection of miR‐195 overexpression or inhibitor lentiviral vector in mice

The mice were weighed, anaesthetized with 2% avertin solution. Then the mice were fixed on the operating table, muscles were separated, the chest was opened between the third and fourth ribs space on the left, the aorta of the heart was exposed, and the aorta was clamped with the artery clip, the ventricular cavity was intraluminally injected with 70 μL of lentivirus containing a final concentration of 10^8^ transducing U/mL of lenti‐miR‐195 or a negative control or lenti‐AMO‐miR‐195, and the arterial clip was removed and sutured. All animal experiments and animal welfare involved have been approved by the Institutional Animal Care and Use Committee of Harbin Medical University, College of Pharmacy (No. IRB3004619); after injection of lenti‐miR‐195 inhibitor for 1 week, the mice were applied TAC surgery; 8 weeks later, the mice were anaesthetized to detect cardiac function and electrocardiogram.

### 2.3 Construction of mouse cardiac hypertrophy model induced by Transverse Aortic Constriction (TAC)

TAC method was used to establish a model of cardiac hypertrophy in mice. The TAC model can simulate haemodynamic overload to cause left ventricular hypertrophy and pathological remodelling. The male C57BL/6 mice weighing 22‐26 g were anaesthetized with 2% avertin with intraperitoneal injection. The mouse was fixed on the operating table in a supine position, the debris in the oral cavity of the mouse was cleaned, the tracheal tube was inserted into the trachea from the oral cavity, and the small animal ventilator was connected. Make a small incision near the end of the sternum, the muscular tissue and glands were carefully separated, use a 26 G cushion needle to gently bypass the 5‐0 ligature line, pass the dead knot, narrow the aortic arch, draw out the cushion needle, and the sternum and skin were immediately sutured with the 6‐0 ligation thread. After the operation, the mice were returned to the animal room for 8 weeks to ensure that the model mouse and the sham operation mice were kept in the same condition. Echocardiography was used to detect whether the model of myocardial hypertrophy was successfully established. In the sham operation group, all treatment methods are the same as the TAC model group except for the aortic arch narrowing operation.

### 2.4 Echocardiographic test

The mice were firstly weighed, then they were generally anaesthetized by intraperitoneal injection of 2% avertin solution at a volume of 0.l mL/10 g of body weight. The mice were then fixed on the supine position on the testing pad. The Echocardiography (Vevo2100, Visualsonics, Canada) was used to detect the changes of cardiac function before and after the establishment of HF model in mice, as described previously.^23^ Echocardiography was used to detect the long and short axis data of the heart, the long axis of the left ventricle near the sternum, the longest left ventricular length, including the mitral and aortic valves and the short axis of the left ventricle near the sternum, which displayed as the arcuate contour of the left ventricle epicardium is the ideal detection field. The long axis of left ventricular and short axis papillary muscle of left ventricular levels were recorded for measuring diastolic left ventricle anterior wall (LVAWd), systole left ventricle anterior wall (LVAWs), left ventricular internal dimension at end‐diastole (LVIDd), left ventricular internal dimension at systole (LVIDs), thickness of diastolic left ventricular posterior wall (LVPWd), thickness of systolic left ventricular posterior wall (LVPWs), ejection fraction (EF) and fractional shortening (FS).

### 2.5 Detection of ventricular arrhythmias

The mice were generally anaesthetized with avertin solution. An 8‐electrode catheter was inserted into the right ventricle, and this procedure has been applied in our previous study (1.1 F, Octapolar EP catheter; SciSense).^23^ A procedural electrical stimulation device (GY6000; HeNanHuaNan Medical Science & Technology Ltd.) was used. The detection electrodes were inserted into the right ventricle from the external jugular vein of the mouse, and data were collected by stimulating specific stimulation frequencies and intensity. The ventricular tachyarrhythmia (VT) was set by applying a series of 10 consecutive electrical pulses, the initial electrical stimulation coupling interval is 80 ms (S1), and followed by two extra stimuli (S2, S3), decreasing in order at a coupling intervals of 2 ms, respectively. Successful induction of VT was defined as the appearance of rapid non‐sinus rhythm ventricular activations lasting for three beats or more.

### 2.6 Electron microscopy

The ventricular tissue was removed from the mouse heart and fixed in glutaraldehyde and maintained for two hours. Sodium cacodylate buffer was used to wash the tissue slices for three times by for 10 minutes each. The ventricular tissue slices were fixed with 1% osmium tetroxide for 1 hours. Then ventricular tissues were dehydrated in 50%, 70%, 90%, 100% ethanol; we use uranyl acetate solution and lead citrate solution to stain primary ventricular tissue slices; and the pathological changes were examined by a JEOL TEM (JEM‐1011; JEOL Ltd., Tokyo, Japan).

### 2.7 Isolation of neonatal mouse ventricular myocytes (NMVMs)

Ventricular myocytes were isolated from the hearts of 1‐day‐old neonatal mice (C57BL/6) and were differentially plated to remove fibroblasts, as described previously.^23^ Briefly, neonatal mouse ventricles were rapidly removed and cut into l to 2 mm^3^, add 0.25% trypsin for heart tissue digestion. Digestion steps were repeated until the tissues disappeared, then collected suspension cells by centrifugation at 2000 g for 5 minutes. DMEM medium was used to culture cells (Biological Industries), which was added with 10% foetal bovine serum (Biological Industries) and supplemented with penicillin (100 U/mL)/streptomycin (100 U/mL; Beyotime); the cells were cultured at 37℃. After fibroblast showed adherence after 120 minutes, the cell suspension which mainly included cardiomyocytes was plated at 3 ~ 5×10^5^ cells per well using DMEM. Add 5‐bromo‐2‐deoxyuridine (10 nM) into the DMEM medium to inhibit fibroblasts proliferation.

### 2.8 Transfection procedures

miR‐195 with or without AMO‐miR‐195, or negative control (NC) siRNAs at a concentration of 100 pmol/mL were transfected into neonatal primary mouse ventricular myocytes using X‐treme GENE siRNA transfection reagent (Roche, Basel, Switzerland, Cat.#04476093001). miR‐195 mimics sequences were shown as following: sense: 5'‐UAGCAGCACAGAAAUAUUGGC‐3'; antisense: 5'‐ CAAUAUUUCUGUGCUGCUAU

U‐3' and miR‐195 inhibitor sequences were shown as following: 5'‐GCCAAUAUUUCUGUGCUGCUA‐3'. 48 hours after transfection, cardiomyocytes were collected to extract total RNA or were used for protein extraction. 10^6^ infectious titre miR‐195 lentivirus vector was also transfected it into the cultured neonatal mouse ventricular myocytes. miR‐195 lentivirus vector was designed to carry green fluorescent protein to make sure it will be successfully estimated the efficiency of miR‐195 lentivirus infection, and the cardiomyocytes image was obtained by microscopy after 48h transfection of miR‐195 lentivirus vector in cardiomyocytes. Sequence of miR‐195, AMO (anti‐microRNA antisense oligodeoxyribonucleotide)‐miR‐195, NC is shown in Table 1.

**Table 1:** Sequence of miR‐195, AMO (anti‐microRNA antisense oligodeoxyribonucleotide)‐miR‐195, NC

Primer names	Sequences
miR‐195 Sense:	5'‐UAGCAGCACAGAAAUAUUGGC‐3'
miR‐195 Antisense:	5'‐ CAAUAUUUCUGUGCUGCUAUU‐3'
AMO‐miR‐195	5'‐ GCCAAUAUUUCUGUGCUGCUA‐3'
NC Sense:	5'‐ UUCUCCGAACGUGUCACGUTT −3'
NC Antisense:	5'‐ ACGUGACACGUUCGGAGAATT −3'

### 2.9 RNA extraction

Total RNA was extracted using the phenol chloride method. Left ventricular (LV) tissues of C57BL/6 mice or primary neonatal cardiomyocytes were washed with diethylpyrocarbonate (DEPC) water‐treated phosphate‐buffered saline (PBS) buffer, then processing with Trizol reagent (Invitrogen). The quality of the extracted RNA samples was confirmed by denaturing gel, and those with clear 28s and 18s bands can be used for subsequent experiments.

### 2.10 Quantitative real‐time PCR (qPCR)

Complementary DNA was synthesized using random primers as shown in manufacturer's instructions (ReverTra Ace qPCR RT Kit, TOYOBO; FSQ‐101). LightCycler 480 SYBR Green I Master (Roche; Cat#4707516001) was used for real‐time PCR. The target genes were quantified on the ABI 7500 fast Real‐Time PCR system (Applied Biosystems). Melting curve of target genes was used to estimate the specificity of our amplified product. Primer Premier 6.0 program (PREMIER Biosoft International, USA) was used to design all PCR primers. The comparative cycle threshold (Ct) method (2^‐ΔΔCt^) was applied to calculate the relative expressions of mRNAs or miR‐195. Each data point of each sample was then normalized to GAPDH or U6, which were recognized as internal reference. The primer sets used in our analyses are shown in **Table** **2** **.**

**Table 2:** The Gene‐specific primers used for real‐time PCR analyses

Primer names	Sequences
miR‐195‐Forward:	CGGGCTAGCAGCACAGAAA
miR‐195‐Reverse:	CAGCCACAAAAGAGCACAAT
U6‐Forward:	GCTTCGGCAGCACATATACTAAAAT
U6‐Reverse:	CGCTTCACGAATTTGCGTGTCAT
ANP‐Forward:	CTCCGATAGATCTGCCCTCTTGAA
ANP‐Reverse:	GGTACCGGAAGCTGTTGCAGCCTA
BNP‐Forward:	CTGGCATACTCTTGCAGCCT
BNP‐Reverse:	CTGCCTTGTGAAGGGGTGAT
β‐MHC‐Forward:	CCAGAAGCCTCGAAATGTC
β‐MHC ‐Reverse:	CTTTCTTTGCCTTGCCTTTGC

### 2.11 Western blot analysis

We extracted the protein samples from heart tissues of C57BL/6 mice or primary cultured cardiomyocytes for western blot detection of related proteins; frozen or fresh tissue was homogenized with protein lysate contained 40% by volume of 10% SDS, 60% RIPA and 1% protease inhibitor Cocktail (Sigma‐Aldrich, P8340‐1 ML). The homogenate was then centrifuged at 12 000 g for 30 minutes and the supernatants (containing cytosolic and membrane fractions) were collected for protein concentration detection, using the BCA kit (Beyotine, P0011) with TECAN Infinite 200 PRO NanoQuant detection system. Protein samples were fractionated by SDS‐PAGE (10% polyacrylamide gels for connexin43) then transferred to nitrocellulose blotting membrane. The primary anti‐Cavβ1 (Abcam, Cat# ab85020) and anti‐Kir2.1 antibody (Alomone Labs, Cat# APC‐026), and anti‐Kv4.3 antibody (Alomone Labs, Cat#APC‐017) were used. GAPDH (Beijing, China, Cat#TA‐08) was selected as an internal control for proteins. Western blot bands were imaged on the Odyssey Infrared Imaging System (LI‐COR Biosciences). The band intensity (area × OD) was measured in each group and normalized to GAPDH with Odyssey v1.2 software.

### 2.12 Immunocytochemistry

Cultured NMVCs were incubated with anti‐Cavβ1 (Cat# ab85020;Abcam, Cambridge, UK) and antibody of Kir2.1 (Alomone Labs, Cat# APC‐026), antibody of Kv4.3 (Alomone Labs, Cat#APC‐017), antibody of α‐actinin (sigma, Cat#A7811) at 4°C refrigerator for overnight. The cells were washed with PBS buffer and incubated with the secondary antibodies (1 hours at room temperature) conjugated to Alexa Fluor 488 or Alexa Fluor 594 (Molecular Probes). The preparations were then examined under an immunofluorescence microscope.

### 2.13 Dual‐Luciferase reporter assay

We cloned fragments from CACNB1 3’UTR region for analysis of the luciferase activity, position 943‐949 on the 3’UTR of CACNB1 contained the predicted putative binding sequences for miR‐195; we also cloned the KCNJ2 3’UTR region, which has miR‐195 putative binding sequences (the position of 395‐401 and 2183‐2190 on the 3’UTR of KCNJ2) and KCND3 3’UTR region, containing the miR‐195 predicted binding sequences (the position of 173‐179 on the 3’UTR of KCND3), then they were amplified by PCR, the products were cloned into the pSICHECK‐2‐control vector. Mutagenesis nucleotides were also designed at different binding site. The sequence of miR‐195 mimic is 5’‐ UAGCAGCACAGAAAUAUUGGC‐3’ (synthesized based on the sequence of mmu‐miR‐195 (miRBase Accession No. MIMAT0000225); that of NC is 5’‐UUCUCCGAACGUGUCACGUAA‐3’; the sequence of the antisense 2’‐O‐methyl (2’‐O‐Me) oligonucleotide for miR‐195 is 5’‐ GCCAAUAUUUCUGUGCUGCUA‐3’, HEK293T cells were transfected with 0.5μg psi‐CHECK^TM^‐2‐target DNA with lipofectamine 2000 (Invitrogen) and supplemented with 20 μM/LmiR‐195, AMO‐miR‐195, or Nc. After transfection for 48h, Firefly and renilla luciferase activities were determined by luciferase assay kits (Promega, Cat.#E1910) as indicated by relative luminescence units (RLU), and luminometer (GloMax^TM^ 20/20, Promega) was recorded according to the manufacturer's instructions.

### 2.14 Statistical Analysis

ALL experimental data were described as MEAN ± SEM. The two‐tailed Student's *t* test was applied for comparisons between the two groups. One‐way ANOVA was used in multi‐group's comparisons were performed for multiple pairwise comparisons. The χ^2^ test is used to compare nonparametric data set comparisons. SPSS19.0 software was used for all statistical analyses. *P < *.05 was considered as statistical significance.

## 3 RESULTS

### 3.1 Increased expression of miR‐195 and Ion channel Remodelling in the model of cardiac hypertrophy induced by TAC

**Figure 1:**
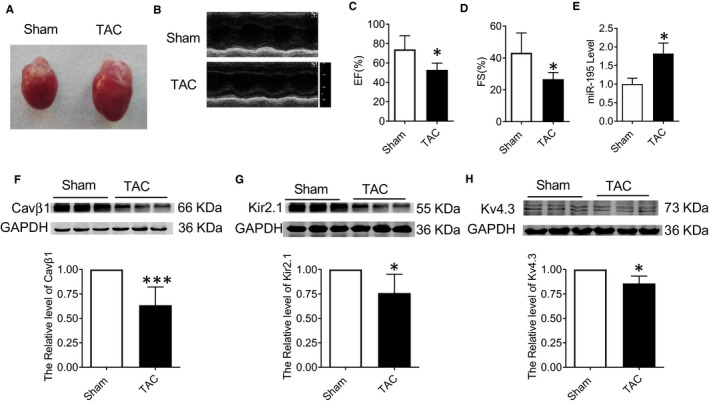
Increased expression of miR‐195 in the model of cardiac hypertrophy induced by TAC. (A‐D) Cardiac function was decreased in cardiac hypertrophy induced by TAC compared with sham‐operated group, EF (%) and FS (%) values were significantly decreased in TAC group, *P < .05 vs. Sham, n = 6; (E) real‐time PCR analysis showing the expression of miR‐195 was increased in myocardium of cardiac hypertrophy induced by TAC compared with sham‐operated group,*P < .05 vs. Sham, n = 7; (F‐H) Western blot analysis of Cavβ1, Kir2.1 and Kv4.3 in the cardiac hypertrophy model. The protein levels of Cavβ1 were found to be significantly reduced in myocardium of cardiac hypertrophy induced by TAC, ***P < .001 vs. Sham, n = 7. The protein expressions of Kir2.1 were significantly decreased in myocardium of cardiac hypertrophy induced by TAC, *P < .05 vs. Sham, n = 6. Western blot analysis showing significant down‐regulation of Kv4.3 in myocardium of cardiac hypertrophy induced by TAC, *P < .05 vs. Sham, n = 3

Cardiac hypertrophy mouse model was first established by transverse aortic constriction (TAC). Eights weeks later, echocardiography was performed on the mouse heart (Figure 1A,B). Compared with the sham‐operated group, the cardiac function was significantly impaired with the decreased EF (%) and FS (%) in the TAC‐induced hypertrophy model group (Figure 1C,D, *P < .05 vs. Sham), which indicated that the mouse cardiac hypertrophy model was successfully established. The miR‐195 level was detected by real‐time PCR. Compared with the sham operation group, miR‐195 showed significant increase in the myocardial tissue of the TAC‐induced hypertrophy model group (Figure 1E, *P < .05 vs. sham). Compared with the sham group, the protein expressions of Cavβ1 were significantly decreased in myocardium of cardiac hypertrophy induced by TAC (Figure 1F, ***P < .001 vs. Sham), Kir2.1 were significantly decreased in myocardium of cardiac hypertrophy induced by TAC (Figure 1G, *P < .05 vs. Sham), the protein expression of Kv4.3 was significantly decreased in myocardium of cardiac hypertrophy induced by TAC (Figure 1H, *P < .05 vs. Sham).

### 3.2 Overexpression of miR‐195 impaired cardiac function and induced ultrastructure damage in mice

**Figure 2:**
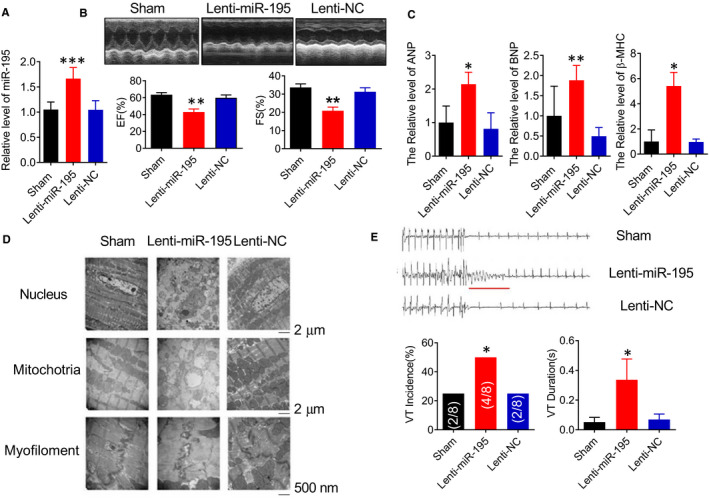
Overexpression of miR‐195 impaired cardiac function, promoted the occurrence of arrhythmias. (A) Compared with the NC‐operated group, overexpression of miR‐195 carried by lentivirus significantly increased the level of miR‐195 in the heart determined by real‐time PCR, ***P < .001 vs. NC, n = 5. (B) Upper: Representative M‐mode echocardiographs from sham control and lenti‐miR‐195, NC mice. Lower: Compared with the sham‐operated group, the value of EF (%) and FS (%) in the Lenti‐miR‐195 injection group was significantly decreased, **P < .01 vs. NC, n = 6. (C) Compared with the sham‐operated group, the expressions of ANP (*P < .05 vs. NC, n = 5), BNP (**P < .01 vs. NC, n = 5), β‐MHC (*P < .05 vs. NC, n = 4) were significantly increased after miR‐195 treatment. (D) Overexpression of miR‐195 impaired ultrastructure in mice. Compared with sham‐operated group, overexpression of miR‐195 can induce the nucleus impairment and swollen mitochondria of the cardiomyocytes, the myofilament was fractured, intercalated disc was cracked. Magnification 10 000 times and 30 000 times. (E) Overexpression of miR‐195 promoted the occurrence of arrhythmias. Upper: The representative map of procedural electrical stimulation. Lower: Pro‐arrhythmic effects of miR‐195 in healthy mice. A prominent finding here is that overexpressing miR‐195 was sufficient to induce VT in healthy hearts. Compared with sham‐operated group, the occurrence of arrhythmia in overexpressing miR‐195 group was significantly increased, and the duration was significantly prolonged, *P < .05 vs. NC, n = 8 ‐ 12

The next step we want to know whether miR‐195 shows a key role in triggering cardiac hypertrophy, and whether miR‐195 overexpression can induce cardiac dysfunction. To test our hypothesis, the left ventricle was injected with lenti‐pre‐miR‐195 by aortic clipping. The miR‐195 was detected by PCR at 4 weeks after lentivirus injection. Compared with NC group, lenti‐miR‐195 significantly increased the expression level of miR‐195 (Figure 2A, ***P < .001 vs. NC). Because miR‐195 lentivirus vector was designed to carry green fluorescent protein to make sure it will be successfully estimated the efficiency of miR‐195 lentivirus infection in cardiomyocytes, the cardiomyocytes image with transfection of miR‐195 lentivirus vector was shown in Figure S1. These data further confirmed the successful transduction of miR‐195 into cardiomyocytes. miR‐195 overexpression impaired cardiac function in mice. Compared with the NC group, the value of EF (%) and FS (%) in the lenti‐miR‐195 injection group was significantly decreased (Figure 2B, **P < .01 vs. NC). The other cardiac functional parameters such as LVID;d, LVID;s, LVAW;d, LVAW;s, LVPW;d, LVPW;s were shown in Table 3. After over expression of miR‐195, the cardiac hypertrophy‐related index atrial natriuretic peptide (ANP), brain natriuretic peptide (BNP) and β‐MHC mRNAs levels were significantly higher (Figure 2C, *P < .05, **P < .01 vs. NC). These data indicate that cardiac‐specific overexpression of miR‐195 in mice induces heart failure. The effect of miR‐195 on the ultrastructure of myocardium was detected by transmission electron microscopy. The results showed that in the NC group, we could not observe any morphological changes, the nucleus was intact, the mitochondria were average cross‐sectionally arranged in a compact state, and the myofilament was intact. Compared with the NC group, the nucleus and mitochondria were swollen and deformed, and the mitochondria were paralysed in miR‐195 overexpression group. There is a fuzzy dissolution phenomenon; the myofilament connection is disordered or the fracture is increased; and the disc is cracked in miR‐195 over expression group (Figure 2D). miR‐195 induced pathological cardiac remodelling in mice.

**Table 3:** Cardiac function parameters in miR‐195 overexpression mice

	LVID;d	LVID;s	LVAW;d	LVAW;s	LVPW;d	LVPW;s
Sham	3.31 ± 0.36	2.20 ± 0.32	1.09 ± 0.13	1.66 ± 0.14	0.69 ± 0.15	0.91 ± 0.22
Lenti‐miR‐195	3.65 ± 0.48	2.89 ± 0.45^*^	1.05 ± 0.29	1.34 ± 0.27^*^	0.67 ± 0.17	0.79 ± 0.16
Lenti‐NC	3.58 ± 0.38	2.47 ± 0.40	1.04 ± 0.15	1.47 ± 0.11	0.58 ± 0.19	0.83 ± 0.28

Note

LVID;d: left ventricular internal dimension at end‐diastole; LVID;s: left ventricular internal dimension at systole; LVAW;d: diastolic left ventricle anterior wall; LVAW;s: systole left ventricle anterior wall; LVPW;d: thickness of diastolic left ventricular posterior wall; LVPW;s: thickness of systolic left ventricular posterior wall.

^*^ *P* < .05 vs. NC

### 3.3 miR‐195 overexpression promotes the occurrence of arrhythmia in mice

In our previous study, the incidence of ventricular tachycardia (VT) and prolongation of VT duration were significantly increased induced by programmed left ventricular tachypacing in HF mice.^23^ After induction of arrhythmia by TAC, expression of miR‐195 was increased. We observed the effect of miR‐195 on arrhythmia by injection of miR‐195 overexpression lentivirus vector in vivo. Four weeks after overexpression of miR‐195, the mice were subjected to programmed left ventricular tachypacing. Our results showed that compared with the NC group, the probability of arrhythmia induction was significantly increased in the lenti‐miR‐195 injection group, and the duration of arrhythmia was significantly prolonged (Figure 2E, *P < .05 vs. NC). These data indicate that the likelihood of arrhythmia induction was increased by miR‐195 overexpression in normal mice.

### 3.4 Lenti‐miR‐195 inhibitor improved cardiac function and reduced the occurrence of arrhythmia in cardiac hypertrophy induced by TAC in mice

**Figure 3:**
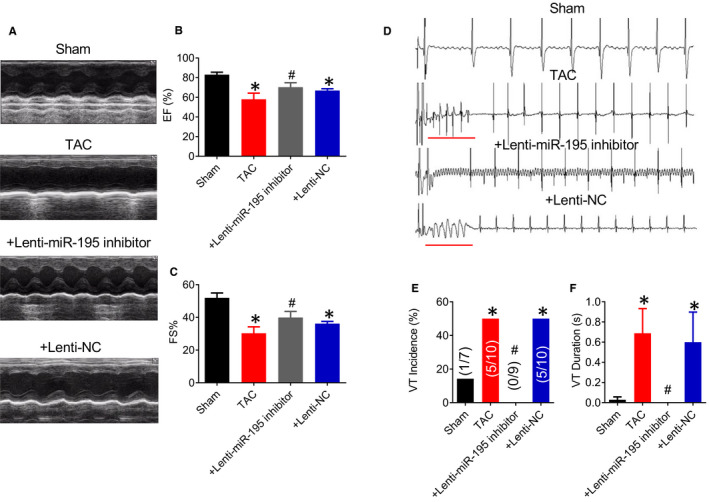
AMO‐miR‐195 improves cardiac function and arrhythmia in cardiac hypertrophy mice. (A‐C) Compared with the Sham group, the value of EF (%) and FS (%) in the TAC group and TAC + Lenti‐NC (+Lenti‐NC) group was significantly decreased, *P < .05 vs. Sham, n = 6. The values of EF (%) and FS (%) were reversed in the TAC + Lenti‐mir‐195 inhibitor (+Lenti‐mir‐195 inhibitor) group compared with the + Lenti‐NC group, #*P* < .05 vs. +Lenti‐NC, n = 6. (D‐F) Compared with the sham‐operated group, the incidence of arrhythmia in mice was significantly increased, and the duration of arrhythmia was significantly prolonged in TAC‐induced cardiac hypertrophy, which were reversed in the + Lenti‐mir‐195 inhibitor group compared with the + Lenti‐NC group (*P < .05 vs. Sham, n = 9, #*P* < .05 vs. +Lenti‐NC, n = 10)

One week after injection with Lenti‐miR‐195 inhibitor or negative control lentivirus, the mice were divided into Sham group, TAC group, TAC + miR‐195 inhibitor group (+Lenti‐mir‐195 inhibitor) and TAC + NC (+Lenti‐NC) group. After 8 weeks, the ultrasound ejection fraction (EF%) and short axis shortening rate (FS%) of each group of mice were detected by echocardiography (Figure 3A). Compared with the Sham group, the EF and FS values of the TAC group and + Lenti‐NC group were significantly decreased (Figure 3B,C *P < .05 vs. Sham), which were reversed by + Lenti‐mir‐195 inhibitor (#*P* < .05 vs. +Lenti‐NC). The other cardiac functional parameters such as LVID;d, LVID;s, LVAW;d, LVAW;s, LVPW;d, LVPW;s were recorded in our study as shown in Table 4. The programmed electrical stimulation was used to detect the occurrence of arrhythmia. Compared with the sham‐operated group, the incidence of arrhythmia and prolonged induction duration of arrhythmia were increased in TAC‐induced hypertrophy model group (Figure 3D‐F, *P < .05 vs. Sham). Compared with mice in the + Lenti‐NC group, the incidence of arrhythmias and the induction duration of arrhythmia in the + Lenti‐mir‐195 inhibitor group were reduced (Figure 3D‐F, #*P* < .05 vs. +Lenti‐NC).

**Table 4:** Cardiac function parameters in TAC and miR‐195 inhibitor treatment mice

	LVID;d	LVID;s	LVAW;d	LVAW;s	LVPW;d	LVPW;s
Sham	3.32 ± 0.44	1.62 ± 0.45	1.07 ± 0.14	1.76 ± 0.22	0.92 ± 0.14	1.57 ± 0.21
TAC	3.34 ± 0.34	2.32 ± 0.27^*^	0.92 ± 0.17	1.30 ± 0.21^*^	1.15 ± 0.31	1.51 ± 0.55
+Lenti‐miR‐195 inhibitor	3.66 ± 0.49	2.23 ± 0.63^#^	1.03 ± 0.26	1.62 ± 0.30^#^	1.09 ± 0.29	1.62 ± 0.32
+Lenti‐NC	3.42 ± 0.17	2.18 ± 0.18^*^	0.96 ± 0.10	1.45 ± 0.16	1.15 ± 0.24^*^	1.61 ± 0.27

Note

+Lenti‐miR‐195 inhibitor: TAC + Lenti‐miR‐195 inhibitor; +Lenti‐NC: TAC + Lenti‐NC; LVID;d: left ventricular internal dimension at end‐diastole; LVID;s: left ventricular internal dimension at systole; LVAW;d: diastolic left ventricle anterior wall; LVAW;s: systole left ventricle anterior wall; LVPW;d: thickness of diastolic left ventricular posterior wall; LVPW;s: thickness of systolic left ventricular posterior wall.

a ^*^ *P* < .05 vs.Sham.

b ^#^ *P*< .05 vs.TAC.

### 3.5 miR‐195 inhibits the protein expression of Cavβ1, Kir2.1, Kv4.3 in vivo

**Figure 4:**
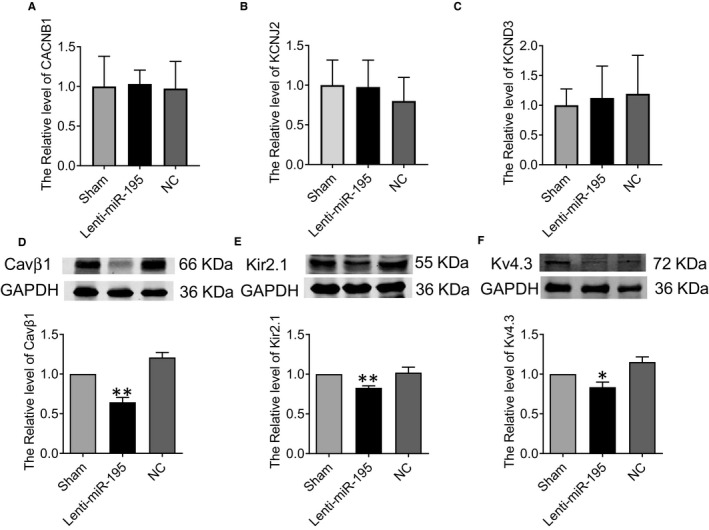
miR‐195 inhibits the protein expression of Cavβ1, Kir2.1 and Kv4.3 in vivo. (A‐C) qPCR showed the changes of CACNB1/KCNJ2/KCND3 transcripts in cardiac tissues from overexpressing miR‐195 mice, n = 5‐9. (D) Verification of the specificity of miR‐195 on Cavβ1. Compared with NC, the expression of Cavβ1 protein in the overexpressed miR‐195 group was decreased. **P < .01 vs. NC, n = 5. (E) Compared with NC, the expression of Kir2.1 protein in the overexpressed miR‐195 group was decreased. **P < .01 vs. NC, n = 6. (F) Compared with NC, the expression of Kv4.3 protein in the overexpressed miR‐195 group was decreased.*P < .05 vs. NC, n = 3

To assess whether the miR‐195‐triggered cardiac arrhythmia is dependent on its inhibiting roles in ion channels, we detected the expression of calcium and potassium channel. Because we predicted that miR‐195 can interact with the 3’UTR of KCNJ2 encoding Kir2.1 protein, 3’UTR of CACNB1 encoding Cavβ1 protein and 3’UTR of KCND3 encoding Kv4.3 by Targetscan bioinformatics website. The CACNB1/KCNJ2/KCND3 mRNA levels were not changed (Figure 4A‐C). To investigate the regulatory role of miR‐195 on Cavβ1, Kir2.1 and Kv4.3 protein expression, we used Western blot to detect Cavβ1, Kir2.1 and Kv4.3 in mouse hearts after lenti‐miR‐195 treatment. Compared with NC group, Cavβ1 (Figure 4D, **P < .01 vs. NC), Kir2.1 (Figure 4E, **P < .01 vs. NC) and Kv4.3 (Figure 4F, *P < .05 vs. NC) were down‐regulated in miR‐195 overexpression group (Figure 4).

### 3.6 miR‐195 overexpression increased ANP, BNP and β‐MHC expression in cultured neonatal cardiomyocyte of mice

**Figure 5:**
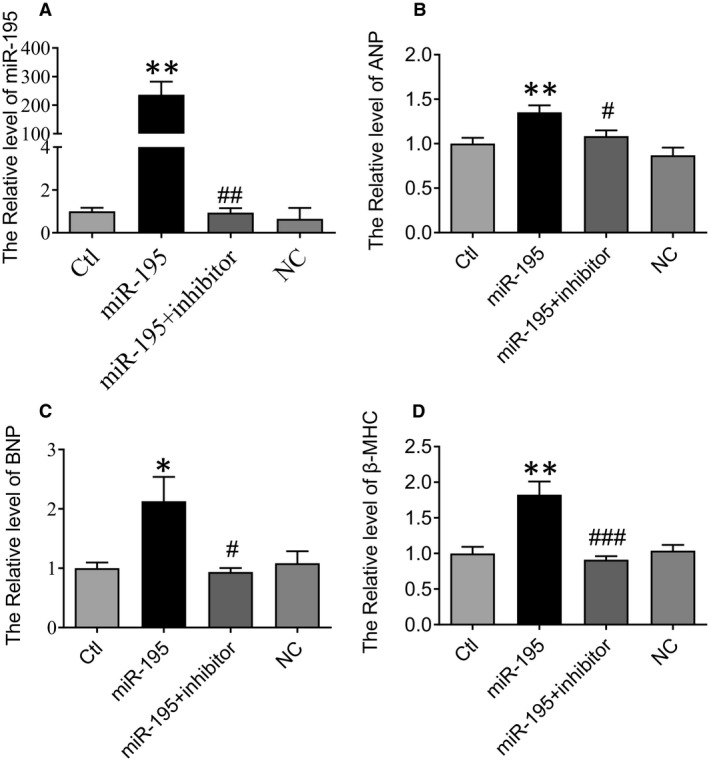
Overexpression of miR‐195 increased ANP, BNP and β‐MHC expression in cultured neonatal cardiomyocyte of mice. (A) Verification of uptake of miR‐195 by cultured neonatal cardiomyocyte after transfection. Compared with NC group, the expression level of miR‐195 was significantly increased in cardiomyocyte of miR‐195 mimics group, **P < .01 vs. NC, n = 6, which was reversed by miR‐195 inhibitor, ##*P* < .01 vs. miR‐195 mimics, n = 6. (B‐D) Transcripts for ANP, BNP and β‐MHC were detected by real‐time PCR in miR‐195 or/and miR‐195 inhibitor treatment, n = 4‐9. Compared with the NC group, the expression of ANP, BNP and β‐MHC was significantly increased after miR‐195 treatment, *P < .05 vs. NC, **P < .01 vs. NC, n = 4‐7, which was reversed by miR‐195 inhibitor, #*P* < .05, ##*P* < .01, ###*P* < .001 vs. miR‐195 mimics, n = 4‐7

The primary neonatal cardiomyocytes of mice were cultured. After 48h, the cultured cardiomyocytes were transfected with miR‐195 mimics, miR‐195 inhibitor or negative control group. After transfection for 48 hours, the RNA was extracted to detect miR‐195 level and the related hypertrophy‐related indicators. The efficacy of miR‐195, AMO‐195 transfection in altering miR‐195, was detected and miR‐195 expression was detected by real‐time PCR experiments (Figure 5A). Compared with NC group, miR‐195 expression levels showed elevated after transfection of miR‐195 mimics (Figure 5A, **P < .01 vs. NC), and the expression levels of ANP (Figure 5B, **P < .01 vs. NC), BNP (Figure 5C, *P < .05 vs. NC) and β‐MHC (Figure 5D, **P < .01 vs. NC) were increased in miR‐195‐overexpressing cardiomyocytes. miR‐195 inhibitor reversed the increased expression levels of miR‐195 (Figure 5A, ##*P* < .01 vs. miR‐195 mimics) and cardiac hypertrophy‐related indicators, ANP (Figure 5B, #*P* < .05 vs. miR‐195 mimics), BNP (Figure 5C, #*P* < .05 vs. miR‐195 mimics), β‐MHC (Figure 5D, ###*P* < .001 vs. miR‐195 mimics) levels.

### 3.7 miR‐195 inhibits the expression of Cavβ1, Kir2.1 and Kv4.3 in neonatal cultured cardiomyocytes by Western blot and immunofluorescence analysis in vitro

**Figure 6:**
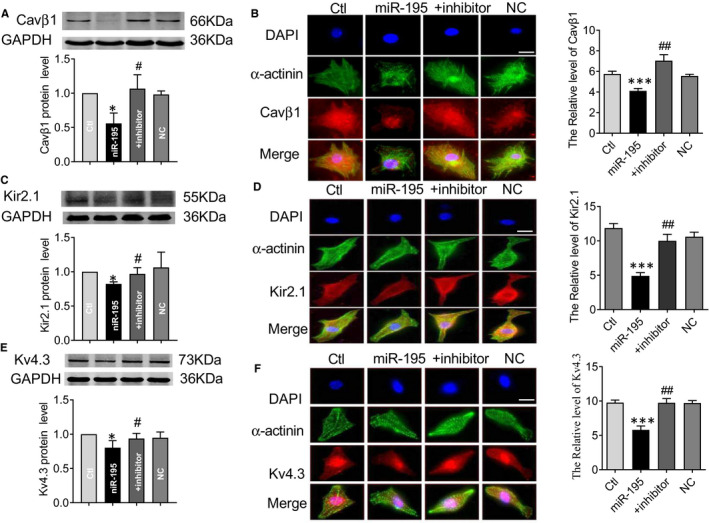
miR‐195 inhibits the expression of Cavβ1, Kir2.1 and Kv4.3 in cardiomyocytes by immunofluorescence and Western blot. (A) Effects of miR‐195 on protein levels of endogenous Cavβ1 in primary cultured cardiomyocytes by Western blot analysis. miR‐195 effectively inhibited the expression of Cavβ1 relative to control group, whereas the scrambled NC miRNA failed to affect the protein levels. In contrast, AMO‐195 rescued the down‐regulation of Cavβ1 elicited by miR‐195. *P < .05 vs. Ctl, #*P* < .05 vs. miR‐195, n = 4. (B) Immunofluorescence experiments were performed to transfect miR‐195 mimics with or without miR‐195 inhibitor and NC in cardiomyocyte. The results showed that compared with the NC group, the expression of Cavβ1 in miR‐195 mimics group was significantly decreased, ***P < .001 vs. NC, n = 6, which was reversed by miR‐195 inhibitor, ##*P* < .01 vs.miR‐195 mimics, n = 6. (C) Effects of miR‐195 on protein levels of endogenous Kir2.1 in primary cultured cardiomyocytes by Western blot analysis. miR‐195 effectively inhibited the expression of Kir2.1 relative to control group, whereas the scrambled NC miRNA failed to affect the protein levels. In contrast, AMO‐195 rescued the down‐regulation of Kir2.1 elicited by miR‐195. *P < .05 vs. Ctl, #*P* < .05 vs. miR‐195, n = 5. (D) Immunofluorescence experiments were performed to transfect miR‐195 mimics with or without miR‐195 inhibitor and NC in cardiomyocyte. The results showed that compared with the NC group, the expression of Kir2.1 in miR‐195 mimics group was significantly decreased, ***P < .001 vs. NC, n = 5, which was reversed by miR‐195 inhibitor, ##*P* < .01 vs. miR‐195 mimics, n = 4. (E) Effects of miR‐195 on protein levels ofen dogenous Kv4.3 in primary cultured cardiomyocytes by Western blot analysis. miR‐195 effectively inhibited the expression of Kv4.3 relative to control group, whereas the scrambled NC miRNA failed to affect the protein levels. In contrast, AMO‐195 rescued the down‐regulation of Kv4.3 elicited by miR‐195. *P < .05 vs. Ctl, #*P* < .05 vs. miR‐195, n = 7. (F) After transfection of cardiomyocytes, immunofluorescence experiments showed that compared with the control group, the expression of Kv4.3 in miR‐195 mimics group was significantly decreased, ***P < .001 vs. NC, n = 8, which was reversed by miR‐195 inhibitor, ##*P* < .01 vs. miR‐195 mimics, n = 4. Magnification 630 times

To investigate the regulatory role of miR‐195 on Kir2.1 and Kv4.3 protein expression in vitro, the protein levels of Kir2.1 and Kv4.3 were detected by Western blot in neonatal cultured cardiomyocytes treated with miR‐195 mimics, with or without miR‐195 inhibitor. The results showed that Cavβ1 (*P < .05 vs. NC), Kir2.1 (*P < .05 vs. NC) and Kv4.3 (*P < .05 vs. NC) protein levels were significantly decreased in miR‐195‐overexpressing cardiomyocytes compared with NC‐treated cell. After the addition of the miR‐195 inhibitor, the down‐regulation of Cavβ1 (#*P* < .05 vs. miR‐195 mimics), Kir2.1 (#*P* < .05 vs. miR‐195) and Kv4.3 (#*P* < .05 vs. miR‐195) was restored to normal level (Figure 6A,C,E). Immunofluorescence experiments were performed to transfect miR‐195 mimics with or without miR‐195 inhibitor and NC in neonatal cultured cardiomyocytes. The expression of Cavβ1, Kir2.1 and Kv4.3 in miR‐195 mimics group was significantly decreased compared with NC group (Figure 6B,D,F, ***P < .001 vs. NC), which were improved by miR‐195 inhibitor (Figure 6B,D,F, ##*P* < .01 vs. miR‐195 mimics).

### 3.8 Direct interaction between miR‐195 and Cavβ1, Kir2.1, Kv4.3 by luciferase assay analysis

**Figure 7:**
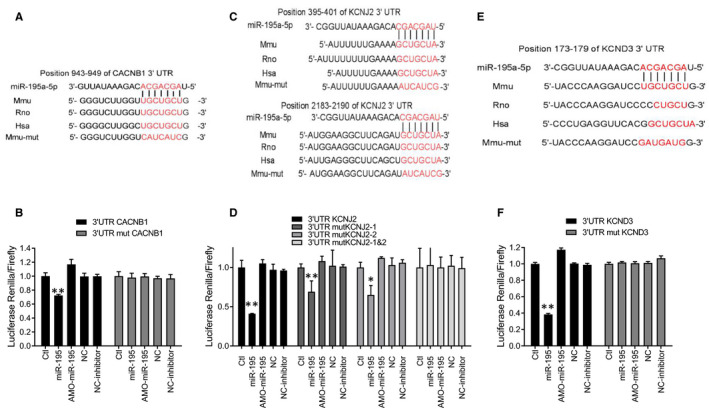
Direct interaction between miR‐195 and Cavβ1, Kir2.1 and Kv4.3. (A) Direct interaction between miR‐195 and Cavβ1. A fragment of miR‐195 that binds to CACNB1. miR‐195 is complementary to the CACNB1 gene 943‐949, which encodes Cavβ1, and the corresponding mutant sequence is designed based on the binding site. (B) Luciferase reporter with a CACNB1 fragment capable of binding to miR‐195. The gene was co‐transfected with miR‐195 into HEK293 cells, and miR‐195 reduced the activity of the luciferase reporter gene, **P < .01 vs. NC, n = 3 batches of cells in each group. The activity of the luciferase reporter gene can be restored by mutating the binding site. (C) miR‐195 seed sequence complementary with the 3’UTR of KCNJ2 predicted by a computational and bioinformatics‐based approach using the Targetscan. miR‐195 is complementary to the 395‐401 and 2183‐2190 sites of the KCNJ2 gene encoding Kir2.1 protein, and the corresponding mutant sequence is designed according to the binding site. (D) miR‐195 reduced the activity of the luciferase reporter gene, **P < .01 vs. NC, n = 3 batches of cells in each group. A single site of mutation was co‐transfected into HEK293 cells with miR‐195, and miR‐195 was still able to reduce the activity of the luciferase reporter gene, **P < .01 vs. NC, n = 3 batches of cells in each group, *P < .05 vs. NC, n = 3 batches of cells in each group. Simultaneous mutation of two binding sites restores the activity of the luciferase reporter gene. (E) A fragment of miR‐195 that binds to KCND3. miR‐195 is complementary to the KCND3 gene 173‐179, which encodes Kv4.3, and the corresponding mutant sequence is designed based on the binding site. (F) The gene was co‐transfected with miR‐195 into HEK293 cells, and miR‐195 reduced the activity of the luciferase reporter gene, **P < .01 vs. NC, n = 3 batches of cells in each group. The activity of the luciferase reporter gene can be restored by mutating the binding site

Our research has confirmed that miR‐195 overexpression promotes the likelihood of cardiac arrhythmia and cardiac hypertrophy, and we went on to clarify the miR‐195‐targeted genes really takes important roles in arrhythmia. As predicted using computational analysis, miR‐195 is complementary to the 943‐949 site of the CACNB1 gene encoding the Cavβ1 protein (Figure 7A).The corresponding vector construct including position 943‐949 on the 3’UTR of CACNB1 was designed, and then the relationship between miR‐195 and CACNB1 was verified by a dual‐luciferase gene detection system. miR‐195 decreased the luciferase activity compared with NC (Figure 7B, **P < .01 vs. NC). However, after mutation binding sites, miR‐195 restored the luciferase activity to normal level (Figure 7B), suggesting that miR‐195 directly regulates Cavβ1. Retrieval results of microRNA targets database showed that miR‐195 was predicted to target KCNJ2 3’UTR. We next clarified whether the position of 94‐100 of KCNJ2 3’UTR, with high conservation, is the potential binding sites of miR‐195 (Figure 7C). Through targetscan software, we predicted that miR‐195 can interact with the 395‐401 and 2183‐2190 of the KCNJ2 encoding the Kir2.1 protein (Figure 7C). Therefore, we designed three different constructs, a mutated first site vector construct, a mutated second site vector construct and a simultaneous mutated two binding site vector constructs. The dual‐luciferase gene detection system was used to verify the relationship between miR‐195 and KCNJ2. miR‐195 significantly decreased the luciferase activity compared with the NC group (Figure 7D, **P < .01 *vs*. NC). After mutating a single site, the luciferase activity in the miR‐195 group was still significantly decreased (Figure 7D, *P < .05 vs. NC). Luciferase activity was restored after mutation of both binding sites (Figure 7D), suggesting that miR‐195 directly targets on the Kir2.1 through two different sites. Through the bioinformatics website, we also predicted that miR‐195 is complementary to the 173‐179 site of the KCND3 gene, which encoded the Kv4.3 (Figure 7E). MiR‐195 significantly decreased the luciferase activity compared with NC (Figure 7F, **P < .01 vs. NC). However, after mutation binding sites, luciferase activity shows similar in NC group (Figure 7F), suggesting that miR‐195 directly targets on Kv4.3. These data indicate that CACNB1/KCNJ2/KCND3 is potential specific targets of miR‐195.

## 4 DISCUSSION

In this study, we identified the expression of miR‐195 was increased in cardiac hypertrophy. miR‐195 is involved in cardiac hypertrophy‐induced arrhythmias by inhibiting Cavβ1, Kir2.1 and Kv4.3 proteins, which were supported by the following data: (1) the expression of miR‐195 was significantly increased in the myocardium of HF mice. The protein expressions of Cavβ1, Kir2.1 and Kv4.3 were decreased in the myocardium of HF mice compared with sham group. (2) Overexpression of miR‐195 by Lenti‐miR‐195 decreased the cardiac function in WT mice and increased the likelihood of arrhythmia induction and duration of arrhythmia in normal mice. (3) Lenti‐miR‐195 inhibitor can reverse the decreased cardiac function, the increased incidence of arrhythmia and prolonged duration of arrhythmia induced by TAC in mice. (4) The protein expressions of Cavβ1, Kir2.1 and Kv4.3 were decreased in mice after miR‐195 treatment. (5) Luciferase assay results showed direct interaction between miR‐195 and Cavβ1, Kir2.1 and Kv4.3.

The reason why miR‐195 was selected for our study has been documented to play key role in pathogenesis and progression of cardiac hypertrophy.^18^ Studies have used isoproterenol to treat rat primary neonatal cardiomyocytes and induce cardiomyocyte hypertrophy models. miR‐195 was significantly increased in the development of cardiac hypertrophy by inhibiting high mobility group protein (HMGA1).^21^ MiR‐195 was up‐regulated in hypertrophic states triggered by pressure overload.^24^ Thus, the results of several research groups have shown that miR‐195 level showed increased in cardiac hypertrophy. Furthermore, it has been confirmed by studies that miR‐195 plays a sufficient role in triggering cardiac hypertrophy or even leading to heart failure using miR‐195 transgenic mice.^20^ Elevated miR‐195 levels in failing myocardium regulate acetylation of pyruvate dehydrogenase (PDH) and ATP synthase, further suppressed SIRT3 and inhibit enzymatic, providing novel pathway for research of miR‐195 directly.^25^ The same conclusions as in the previous literature were also obtained in our study. First, we verified a significant increase in miR‐195 expression in a TAC‐induced cardiac hypertrophy model. In order to identify the role of miR‐195 in cardiac hypertrophy, we constructed miR‐195 overexpression lentivirus and designed and synthesized miR‐195 mimics and their inhibitors, which were verified both in vivo and vitro. Both the animal and cellular results showed that the expression of cardiac hypertrophy (ANP, BNP, β‐MHC) was increased after overexpression of miR‐195. Overexpression of miR‐195 can induce cardiac dysfunction and trigger the susceptibility of arrhythmia. Our study found that inhibition of miR‐195 has the function of maintaining normal cardiac conduction function, reducing the occurrence of arrhythmia in cardiac hypertrophy mice, and improving cardiac function, suggesting that inhibition of miR‐195 in a hypertrophic heart could attenuate the vulnerability to arrhythmia. All these data identified the roles of miR‐195 in triggering arrhythmia accompanied with hypertrophy.

In cardiac pathological hypertrophy, with the increasing degree of hypertrophy, the incidence of malignant arrhythmia is significantly increased, and cardiac electrophysiological remodelling is the main cause of malignant arrhythmia.^1^, ^26^ Electrophysiological remodelling in cardiac hypertrophy is mainly manifested by delayed repolarization, prolongation of QT interval and APD, which increases the dispersed repolarization process.^27^ Studies have shown that prolonged APD is mainly due to disturbed various potassium channels in the pathological state of cardiac hypertrophy.^28^ Potassium channels play a key role in repolarization and arrhythmias induced by cardiac hypertrophy.^29^ A large number of studies have pointed out that in a variety of cardiac hypertrophy or heart failure models, the instantaneous outward potassium current (I_to_), current density decline, is the most stable electrical remodelling feature.^30^ In the case of cardiac hypertrophy, the inward rectifier potassium current (I_K1_) is decreased, the delayed rectifier potassium current (I_Ks_) is down‐regulated, and the weakening of the potassium current causes repolarization abnormalities of the cardiomyocytes, which may cause or aggravate the occurrence of malignant arrhythmia.^31^ In addition, L‐type calcium channels were significantly up‐regulated during cardiac hypertrophy or mild cardiac hypertrophy, and L‐type calcium channels were significantly down‐regulated when hypertrophy entered decompensation or converted to heart failure.^32^ In addition to the above changes in ion channels, pathological cardiac hypertrophy sarcoplasmic reticulum calcium ATPase 2 (SERCA2a) expression disorder can also cause changes in cardiac electrophysiological properties. In our study, it was found that after 8 weeks of TAC, the expression of Cavβ1, Kir2.1 and Kv4.3 protein was significantly decreased.

Upon bioinformatics, we predicted that miR‐195 is complementary to the CACNB1 gene encoding the Cavβ1 ion channel protein, the KCNJ2 gene encoding the Kir2.1 ion channel protein, and the KCND3 gene encoding the Kv4.3 ion channel protein. In fact, recent studies have confirmed that many microRNAs can participate in heart rhythms by acting on different ion channel proteins.^33^ Our previous study has shown that miR‐328 expression is increased in a mouse model of atrial fibrillation, directly targeting the regulation of Cav1.2 and Cavβ1, promoting the progression of atrial fibrillation. Decreasing the expression of miR‐328 can improve the progression of atrial fibrillation.^10^ After myocardial infarction, miR‐1 level was elevated, and Kir2.1 and Cx43 protein was inhibited, which slowed cardiac conduction and promotes ischaemic arrhythmia.^34^ The expression of miR‐16 increased in the marginal zone of myocardial infarction, inhibited the expression of Kir2.1 (KCNJ2) ion channel and promoted the occurrence of arrhythmia.^35^ It can be seen that a variety of different microRNAs increase under pathological conditions, negatively regulate the Cavβ1, Kir2.1 and Kv4.3 ion channel proteins, so that their expression decreases, and then participate in different types of arrhythmias. Therefore, our data further identified that miR‐195 can also participate in arrhythmias by acting on Cavβ1, Kir2.1 and Kv4.3 ion channel proteins.

We first constructed cardiac hypertrophy by TAC in mice and confirmed by Western expression that the decreased expressions of Cavβ1, Kir2.1 and Kv4.3 in the TAC group. After transfection of miR‐195 mimics and inhibitors, our results confirmed that miR‐195 overexpression inhibited Cavβ1, Kir2.1 and Kv4.3 protein expression; after adding miR‐195 inhibitor, the down‐regulated proteins were reversed. After confirming that miR‐195 has a significant inhibitory effect on Cavβ1, Kir2.1 and Kv4.3 ion channel proteins, we use luciferase reporter analysis to confirm the direct regulation between Cavβ1, Kir2.1, Kv4.3 and miR‐195. Therefore, to some extent, we have confirmed the hypothesis that the expression of miR‐195 is increased in cardiac hypertrophy, which inhibits Cavβ1, Kir2.1 and Kv4.3 ion channel protein, and accelerates the occurrence and development of arrhythmia.

### 4.1 Significance of our findings

Our results indicate that miR‐195 is elevated in cardiac hypertrophy, which in turn targets ion channels in cardiomyocytes and promotes cardiac arrhythmias. Knocking down of miR‐195 may affect the expression of relative ion channel protein Cavβ1, Kir2.1 and Kv4.3, which means that miR‐195 is expected to provide a therapeutic target to treat cardiac hypertrophy, arrhythmia and delay of sudden cardiac death. In our study, miR‐195 inhibitors may have been developed and applied in cardiac hypertrophy, which is expected to become a cardiac arrhythmia‐induced arrhythmia treatment.

### 4.2 Limitations of our study

Firstly, we have only studied miR‐195 in animal level and cellular level, and we have explored the knockdown of miR‐195 on cardiac function and heart rhythm in both in cardiac hypertrophy; in future study, relevant electrophysiology experiments should be detected. We did not use patch clamp techniques to study the ion channel function and kinetic changes of the target ion channel. Even so, however, it still provides a perspective for electrophysiological remodelling and arrhythmia after cardiac hypertrophy, that is, miR‐195 may be a potential target to treat arrhythmia.

## 5 CONCLUSION

miR‐195 was increased in cardiac hypertrophy induced by TAC, and miR‐195 overexpression plays key roles in triggering cardiac hypertrophy in heart which resulted in increased likelihood of arrhythmia induction in normal mice. miR‐195 inhibited the expression of Cavβ1, Kir2.1 and Kv4.3, which may contribute to the cardiac arrhythmias induced by cardiac hypertrophy. Together, our studies uncover a novel mechanisms that miR‐195 modulates cardiac hypertrophy by regulating electrical remodelling.

## CONFLICT OF INTEREST

The authors declare no conflict of interest.

## AUTHOR CONTRIBUTION

**Lina Xuan:** Conceptualization (equal); Data curation (lead); Formal analysis (equal); Funding acquisition (equal); Investigation (equal); Methodology (lead); Project administration (equal); Resources (equal); Software (equal); Supervision (equal); Validation (equal); Visualization (equal); Writing‐original draft (supporting); Writing‐review & editing (supporting). **Yanmeng Zhu:** Conceptualization (equal); Data curation (equal); Formal analysis (equal); Investigation (equal); Methodology (equal); Project administration (equal); Resources (equal); Software (equal); Supervision (equal); Validation (equal); Visualization (equal); Writing‐original draft (supporting); Writing‐review & editing (supporting). **Yunqi Liu:** Conceptualization (supporting); Data curation (supporting); Formal analysis (supporting); Investigation (supporting); Methodology (supporting); Resources (equal); Software (equal); Supervision (equal); Validation (supporting); Visualization (supporting); Writing‐original draft (supporting); Writing‐review & editing (supporting). **Hua Yang:** Conceptualization (supporting); Data curation (supporting); Formal analysis (supporting); Investigation (supporting); Methodology (supporting); Resources (supporting); Software (supporting); Supervision (supporting); Validation (supporting); Visualization (equal); Writing‐original draft (supporting). **Shengjie Wang:** Conceptualization (supporting); Data curation (supporting); Formal analysis (supporting); Investigation (supporting); Methodology (supporting); Resources (supporting); Software (supporting); Supervision (supporting); Validation (supporting); Visualization (equal). **Qingqi Li:** Data curation (supporting); Formal analysis (supporting); Methodology (supporting); Software (supporting). **Chao Yang:** Data curation (supporting); Formal analysis (supporting); Investigation (supporting); Supervision (supporting). **Lei Jiao:** Conceptualization (supporting); Methodology (supporting); Software (supporting); Supervision (supporting). **Ying Zhang:** Formal analysis (equal); Investigation (supporting); Supervision (supporting). **Baofeng Yang:** Conceptualization (lead); Data curation (equal); Formal analysis (supporting); Funding acquisition (equal); Investigation (equal); Methodology (supporting); Project administration (equal); Resources (equal); Software (supporting); Supervision (equal); Validation (equal); Visualization (supporting); Writing‐original draft (supporting); Writing‐review & editing (supporting). **Lihua Sun:** Conceptualization (lead); Data curation (equal); Formal analysis (equal); Funding acquisition (lead); Investigation (equal); Methodology (equal); Project administration (lead); Resources (equal); Software (equal); Supervision (equal); Validation (equal); Visualization (equal); Writing‐original draft (lead); Writing‐review & editing (lead). Lina Xuan, Yanmeng Zhu, Baofeng Yang and Lihua Sun designed, performed study, supervised all aspects of the research and analysis. Lina Xuan, Yanmeng Zhu and Lihua Sun finalized the manuscript. Lina Xuan, Yanmeng Zhu completed the animal experiments and molecular targets detect. Yunqi Liu, Hua Yang, Shengjie Wang are responsible for in vitro experiments. Chao Yang, Qingqi Li, Lei Jiao, Ying Zhang assisted in research, data analysis and interpretation.

## Supporting information

Fig S1

## Data Availability

The data in the current study are available from the corresponding authors on reasonable request.
