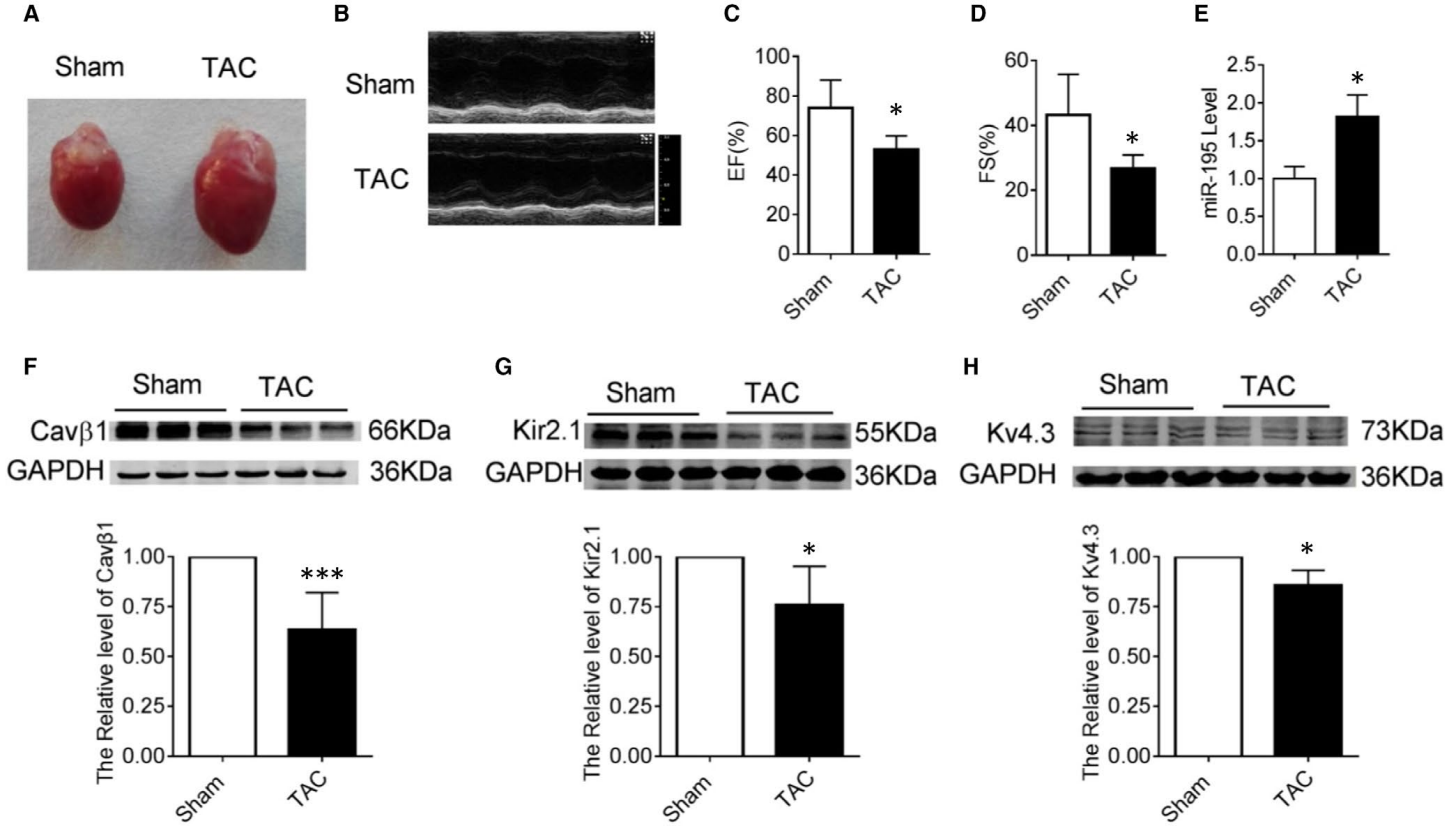# Corrigendum

**DOI:** 10.1111/jcmm.16248

**Published:** 2021-02-10

In Lina Xuan et al,^1^ the published article contains errors in Figure 1. The correct figure is shown below. The authors confirm all results and conclusions of this article remain unchanged.