# Supplementary material for: Up‐regulation of miR‐195 contributes to cardiac hypertrophy‐induced arrhythmia by targeting calcium and potassium channels
Source: J Cell Mol Med. 2020 May 28;24(14):7991–8005. doi: 10.1111/jcmm.15431 (PMC7348160; doi:10.1111/jcmm.15431)
Supplement: Supplementary file 1 — Fig S1 [file JCMM-24-7991-s001.doc]

**Supplementary**

**Up-regulation of miR-195 Contributes to Cardiac Hypertrophy-induced Arrhythmia by Targeting Calcium and Potassium channels**

Lina Xuan^1, #^, Yanmeng Zhu^1^, ^#^, Yunqi Liu^1^, Hua Yang^1^, Shengjie Wang^1^, Qingqi Li^1^, Chao Yang^1^, Lei Jiao^1^, Ying Zhang^1^, Baofeng Yang^1*^, Lihua Sun^1*^

^1^Department of Pharmacology, Harbin Medical University (the State-Province Key Laboratories of Biomedicine-Pharmaceutics of China, Key Laboratory of Cardiovascular Research, Ministry of Education), College of Pharmacy, Harbin Medical University.

^#^These authors contributed equally to this paper.

*Corresponding Authors: Baofeng Yang, yangbf@ems.hrbmu.edu.cn or Lihua Sun: sunlihua0219@163.com; Department of Pharmacology (the State-Province Key Laboratories of Biomedicine-Pharmaceutics of China), Harbin Medical University, 157 Baojian Road, Nangang District, Harbin, Heilongjiang, China 150081; Tel.: +86 451 8667-1354; Fax: +86 451 8667-1354.

Supplementary Figure 1


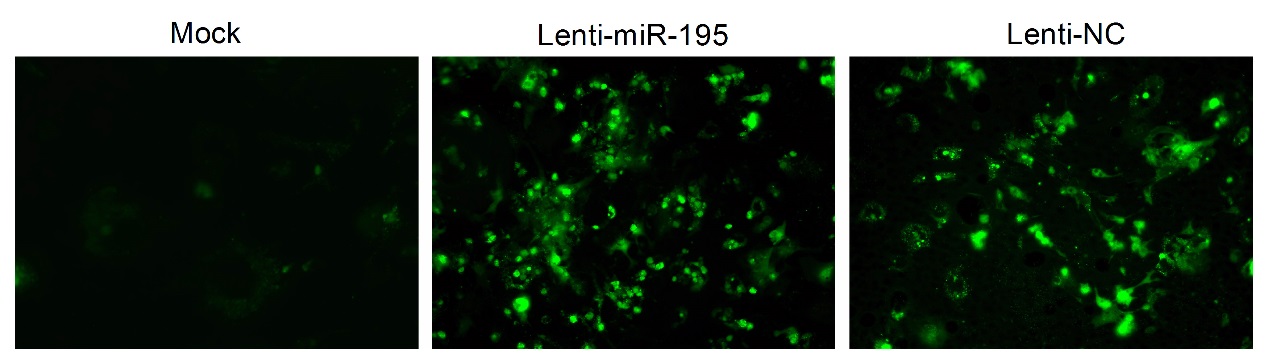


Supplementary Figure 1: The estimated efficiency of miR-195 lentivirus infection in cardiomyocytes. miR-195 lentivirus vector was designed to carry green fluorescent protein, the successful transduction of miR-195 into cardiomyocytes were observed with obvious green fluorescent.
